# Utilization and associated factors of cervical cancer screening service among eligible women attending maternal health services at Adare General Hospital, Hawassa city, Southern Ethiopia

**DOI:** 10.1038/s41598-024-52924-5

**Published:** 2024-02-02

**Authors:** Abiyu Ayalew Assefa, Tihun Feleke, Sintayehu Assefa G/Tsadik, Fekadu Degela, Andualem Zenebe, Geleta Abera

**Affiliations:** 1 Department of Public Health, Hawassa College of Health Sciences, P.O. Box 84, Hawassa, Ethiopia; 2 Hawassa College of Health Sciences, Research and Community Service Directorate, P.O. Box 84, Hawassa, Ethiopia; 3 Department of Midwifery, Hawassa College of Health Sciences, P.O. Box 84, Hawassa, Ethiopia

**Keywords:** Health care, Medical research

## Abstract

This particular study aimed to assess the magnitude of cervical cancer screening service utilization and associated factors among eligible women attending maternal health services at Adare General Hospital, Hawassa city, Southern Ethiopia, 2022. Institution-based cross-sectional study was conducted using a systematic random sampling technique among 299 eligible women from March 1- to April 30, 2022. Data was collected through face-to-face interviews using a pretested structured questionnaire. Data was also entered into Epi-data version 3.1 and exported to SPSS version 25.0 for analysis. Both bivariable and multi-variable logistic regression model was fitted and the presence of association was declared at a *p* value of less than 0.05. The strenth of association was determined using adjusted odd ratios together with a 95% confidence interval. Among interviewed women, 19.8% (95% CI 15.5%, 24.0%) of them had screened for cervical cancer at least once within the past five years. Place of residence (AOR = 0.37; 95% CI 0.14–0.96), modern contraception use (AOR = 2.49; 95% CI 1.04–5.96), discussion about cervical cancer with healthcare providers (AOR = 2.34; 95% CI 1.08–5.07), and comprehensive knowledge about cervical cancer (AOR = 0.25; 95% CI 0.10–0.62) were independently associated with cervical cancer screening service utilization. The study depicted relatively low utilization of cervical cancer screening services. The women were more likely to utilize the service if they are dwellers of urban residences, used modern contraception, had discussion about cervical cancer with healthcare providers, and had good comprehensive knowledge about cervical cancer. Thus, women living in rural areas should get more emphasis on cervical cancer prevention strategies, and improving consistent discussion about cervical cancer with clients visiting for maternal health services by healthcare providers in health facilities also be recommended.

## Introduction

Cervical cancer is the fourth most common cancer among women globally, with an estimated 604 127 new cases and 341 831number of death in 2020^1^. Between 2018 and 2030, it is anticipated that there will be a rise in the number of new instances of cervical cancer from 570,000 to 700,000, as well as an increase in the number of fatalities from 311,000 to 400,000 annually^2^. More than 85% of those afflicted are young, uneducated women who reside in the world’s poorest nations, and the physical, psychological, social, and economic effects of cervical cancer mortality in developing nations of the world are quite significant^3^.

Cervical cancer is the second most frequent disease in Ethiopia, accounting for 9.6% of all cancer cases, and the second cause of cancer death, accounting for 10.3% of all cancer deaths in 2020^4^. Current estimates in Ethiopia, indicate that every year 7445 women are diagnosed with cervical cancer and 5338 die from the disease^5^. Age-standardized death rate was around 16.0 per 100,000 while age-standardized incidence rate was 21.5 per 100,000^4^.

With early detection and treatment, cervical cancer is a condition that is highly preventable and curable. Cervical cancer primary and secondary prevention has been demonstrated to be a successful approach in preventing fatalities linked to it. The World Health Organization (WHO) has highlighted human papillomavirus vaccinations (HPV) and early screening services followed by treatment of precancerous lesions as successful strategies and included them in its recommendations to countries^6^. By achieving the World Health Organization’s 90–70–90 target for cervical cancer, it is thought that these actions will help achieve Sustainable Development Goal target 3.4, which states that “One-third of mortality from non-communicable illnesses in 2030.” The 90–70–90 target aimed to treat at least 90% of all precancerous lesions found during screening by immunizing 90% of all girls by the age of 15, screening 70% of women twice between the ages of 35 and 45, and reducing the age-standardized incidence rate (ASIR) of cervical cancer to less than 4 per 100,000 women globally^7,8^. The World Health Organization advises using the pap smear test, visual examination of the cervix with acetic acid (VIA) or Lugol’s iodine (VILI), and HPV testing as the various screening techniques. When used as a primary screening procedure in low resource settings, VIA is very efficient and reasonably priced^3^.

In most poor countries, efforts to reduce cervical cancer-related mortality are hampered by low screening service coverage and low women’s uptake^9,10^. The federal government of Ethiopia lunched a national cancer control plan through ministry of health which aimed to increase awareness to 50% among the general population and health care providers of early signs and symptoms and opportunities for early detection of the top two cancers (breast and cervical cancer), 80% coverage of vaccination against HPV each year for girls aged 9–13, and 80% coverage of VIA to detect precancerous cervical lesions among non-symptomatic women aged 30–49 every 5 years that are free of charge by 2020. However, the key challenges to implementing the strategy across the country, including the context of our study, are inadequate infrastructure, low awareness, late arrival for screening, and a lack of skilled human resources^11^. Cervical cancer utilization levels of 13.46%^12^, 18.17%^13^, and 22.9%^14^ were reported in several research carried out in various parts of Ethiopia. The likelihood that women would use a cervical cancer screening service depended on their marital status, knowledge of cervical cancer and screening, history of multiple sexual partners, perception of their susceptibility to the disease, consultation with a healthcare provider, educational level, access to information from a health professional, and attitude toward cervical cancer and screening^12–15^.

Ethiopia has insufficient knowledge on cervical cancer, including the disease, attitudes toward it, and screening practices. The ministry of health will be assisted in developing a cervical cancer screening promotion strategy to reduce late diagnosis of cervical cancer disease by identifying the factors related with cervical cancer screening. Also, it aids in the prevention of cervical cancer, which is a useful strategy for a country like Ethiopia that does not have comprehensive access to HPV vaccination. Therefore, the purpose of this study was to assess the magnitude of cervical cancer screening service utilization and associated factors among eligible women attending for maternal health service at Adare General Hospital, Southern Ethiopia.

## Methods

### Study design, study period, and setting

Institution-based cross-sectional study was conducted at Adare General Hospital from March 1- to April 30, 2022. Adare General Hospital is found in Hawassa city, Southern Ethiopia and provides both preventive and curative service since 2011. According to the Sidama regional state base plan estimate, the hospital’s catchment area has an estimated population of 1,368,341 in 2021. The hospital has been gradually expanding the number of beds and medical services it could provide. The hospital provided health care services for 223,425 patients overall in 2016–17; serving for 106,168 and 5103 in out-of-patient and in-patient settings, respectively. According to the hospital’s report, there are 495 employees working there, of which 312 are healthcare professionals and the remaining 183 are support staff (supportive staff). Additionally, six staff members who have received training in cervical cancer screening have done so. Pap smear was utilized prior to the development of visual inspection with acetic acid (VIA) for cervical Ca screening. However, not all eligible women received it on a regular basis. Since 2013, the Hospital has provided premalignant cervical cancer screening using acetic acid visual inspection (VIA) and Cryotherapy services.

### Population

All eligible women (aged 30–49 years according to 2015 guideline for cervical cancer prevention and control in Ethiopia) who came to Adare General Hospital for receiving maternal health service were source population while who came to the hospital for maternal health service during data collection period were considered as study population.

### Eligibility criteria

All eligible women who came to the hospital for maternal health service (F/P service unit, ANC unit, Gynecologic OPD and PNC unit) were included in the study; whereas, women who are unable to hear, had hysterectomy, on laboring and critically ill at the time of data collection period were excluded from the study.

### Sample size determination and sampling procedure

The sample size (n) required for this study was determined using single population proportion formula (n = (Zα/2)^2^ p (1 − p)/d^2^)) by considering the following assumptions; proportion (p) of cervical cancer screening service utilization among women 30–49 years of age at 22.9%, based facility-based cross-sectional study conducted in Hospitals of Wolaita Zone, Southern Ethiopia^14^, 95% confidence interval (Za/2 = 1.96) and margin of error (d = 5%). The calculated sample size was 272. In calculating the sample size, 10% was added to adjust non-response rate and incomplete data. Thus, making the total final sample size to be 299.

The final sample size was allocated to each maternal health service units (family planning unit, antenatal care unit, postnatal unit and gynecologic unit) proportional to their monthly client flow from the comparable quarters of the prior year’s quarterly report. Then, until the necessary sample size was obtained from each unit, eligible study participants were chosen using a systematic random sampling technique. Women between the ages of 30–49 were selected using a sampling interval (k) of 2, with the first eligible woman selected at random.

### Variables of the study

Cervical cancer screening utilization within the past five year was considered as dependent variable (1 for the screening status “Yes”, and 0 for screening status “No”) whereas, socio-demographic and economic factors (age, educational level, average monthly income, place of residence, marital status, occupation), reproductive related factors (parity, age at menarche, abnormal vaginal bleeding, modern contraception use, history of STI, and self-reported HIV serostatus), risk behaviour factors (multiple sexual partners, sexual debut, smoking, chewing chat, alcohol use), knowledge related factors (comprehensive knowledge about CC, knowing someone with CC, and discussion about CC with healthcare provider) were independent variables.

### Data collection instrument and process

Data regarding the socio-demographic information, reproductive health characteristics, knowledge of cervical cancer and behavioral characteristics were collected through face-to-face interview using a structured questionnaire developed by reviewing similar literatures^12–18^ and adapted to fit the objectives of this study. Majority of the questions were closed-ended with pre-coded responses. Two BSc midwives have participated in data collection process.

### Data management and analysis

The completeness and consistency of data values were checked. Paper-based data were entered into Epi-data version 3.1 and exported to SPSS version 25.0 for analysis. Data cleaning was done through running frequencies and coding of data variables was done as appropriate. Descriptive statistics were used to summarize and present the data. Bi-variable and multi-variable logistic regression analyses were used to identify associations between independent and dependent variables. All independent variables in bi-variable logistic analysis having  *p* value of 0.25 or less were selected as a candidate for multi-variable logistic regression analysis. Then multi-variable logistic regression analysis was performed and a *p* value of less than 0.05 was considered to declar statistically significant association. Both crude odds ratio (COR) and adjusted odds ratio (AOR) were computed and interpreted accordingly. The strength of association between variables was determined using AOR  with respective 95% confidence interval.  The goodness-of-fit of model was assessed using Hosmer and Lameshaw test with non-significant (*p* value = 0.069) representing a good fit.

### Data quality assurance

To ensure the quality of the data, 1-day training was given to all the data collectors and supervisors. Before the data collection, a pretest was carried out on 5% of the sample size (in Hawassa Referral Hospital). Based on the findings of the pretest, the necessary modification and correction was made. Initially, questionnaire was developed in English language then it was translated to Amharic language for data collection. Supervision was on daily basis during data collection. Finally, double data entry was performed to check the consistency of the data.

### Operational definitions

#### Cervical cancer screening utilization

In this study cervical cancer screening utilization is proportion of eligible women who received cervical cancer screening at least once within the past five years^19^.

#### Comprehensive knowledge about cervical cancer

In this study, knowledge score was calculated by adding all responses of 15 questions with four main categories: knowledge of risk factors (4 questions); knowledge of symptoms (4 questions); knowledge of prevention mechanism (2 questions); knowledge of methods of cervical cancer screening (2 questions) and knowledge on treatment option of cervical cancer (3 questions). Score one were assigned if respondents were identified correctly while score zero were given if respondents were answered incorrectly or “I don’t know”. Accordingly, the total scores were ranged from 0 to 13. Then, the mean of the total score was calculated to classify level of knowledge. Accordingly, those who have had scored greater than or equal to the mean value was considered as having good comprehensive knowledge about cervical cancer while those who had scored less than the mean was considered to have poor comprehensive knowledge about cervical cancer^19^.

#### Modern contraception use

In this study, use of any form of contraception, including oral contraceptives (pills), injectable contraceptives, implants, or intrauterine devices (IUCD) for more than or equals one month period is considered modern contraceptione use.

### Ethics approval and consent to participate

All the ethical guidelines and principles placed in the Declaration of Helsinki and others, essential to address the ethical aspects of the research commenced in humans were taken into account. Accordingly, ethical approval and clearance was obtained from institution review board of Hawassa College of Health Science. Following these, Hawassa city health department and Adare Hospital were informed on study aim and objective and study permission was obtained. Then a written consent secured from the study subjects through informed consent. The research aim, benefits, and risks were explained to each respondent then data was collected after getting written consent from each respondent. Right of withdrawal from the study was assured at any time during data collection without any penalty. Respondents who did not screened during data collection were counseled about cervical cancer and its screening for better prevention. In order to ensure data confidentiality, no personal identifiers were recorded and codes were used on each questionnaire.

## Results

### Socio-demographic characteristics of respondents

A total of 283 eligible women responded to the questionnaire, giving a response rate of 94.6%. The main reason for missed response rate was lack of co-operation during interview and discarding of questionnaire due to incomplete data.

The mean age of respondents was 35.6 years (± 3.6 SD). Regarding to place of residence majority (63.3%) of respondents were urban dwellers. About 55.8% and 23.0% of respondents were Protestant and Orthodox Christians, respectively, followed by Muslim (19.1%). Only 3.9% of the respondents were single by their marital status. Nearly, half (44.5%) of respondents were housewives in their occupation and 84 (29.7%) of them completed college and above. With regard to average monthly income, about 48.1% of respondents reported greater than or equal to 2000 Ethiopian birr (Table 1).

**Table 1:** Socio-demographic characteristics among eligible women attending maternal health services at Adare General Hospital, Hawassa city, Southern Ethiopia, 2022.

Variables	CCSS utilization		Total, n (%)
	Yes, n (%)	No, n (%)	
Age (in year)			
30–34	28 (50.0)	86 (37.9)	114 (40.3)
35–39	22 (39.3)	94 (41.4)	116 (41.0)
≥ 40	6 (10.7)	47 (20.7)	53 (18.7)
Educational status			
No formal education	15 (26.8)	102 (44.9)	117 (41.3)
Primary school	11 (19.6)	49 (21.6)	60 (21.2)
Secondary school	7 (12.5)	15 (6.6)	22 (7.8)
College and above	23 (41.1)	61 (26.9)	84 (29.7)
Marital status			
Single	6 (10.7)	5 (2.2)	11 (3.9)
Married	38 (67.9)	178 (78.4)	216 (76.3)
Divorced/widowed	12 (21.4)	44 (19.4)	56 (19.8)
Religion			
Protestant	32 (57.1)	126 (55.5)	158 (55.8)
Orthodox	14 (25.0)	51 (22.5)	65 (23.0)
Muslim	10 (17.9)	44 (19.4)	54 (19.1)
Others^@^	0 (0.0)	6 (2.6)	6 (2.1)
Place of residence			
Urban	49 (87.5)	130 (57.3)	179 (63.3)
Rural	7 (12.5)	97 (42.7)	104 (36.7)
Occupational status			
House wife	19 (33.9)	107 (47.1)	126 (44.5)
Self-employed	3 (5.4)	47 (20.7)	50 (17.7)
Governmental employed	16 (28.6)	33 (14.5)	49 (17.3)
Daily laborer	14 (25.0)	20 (8.8)	34 (12.0)
NGO employed	0 (0.0)	12 (5.3)	12 (4.2)
Others^@@^	4 (7.1)	8 (3.5)	12 (4.2)
Average monthly family income (in ETB)			
≥ 2001	33 (58.9)	103 (45.4)	136 (48.1)
1001–2000	17 (30.4)	78 (34.4)	95 (33.6)
≤ 1000	6 (10.7)	46 (20.3)	52 (18.4)

@: Catholic, Gehova witness; @@: Farmer, Student; NGO: Non-governmental organization.

### Reproductive and risk behavior related characteristics of respondents

Most of respondents, 176 (62.2%) had two to four children. Two hundred thirty-three (82.3%) of respondents had first mensuration cycle after age of 12 years and about 213 (75.3%) women experience regular menses. Of all respondents, 193 (68.2%) had used modern contraceptives. Of those, 49 (25.4%) used oral contraceptive pills. Among interviewed women, 120 (42.4%) of them had their first sex at age of below 18 years old. Around 239 (84.5%) of respondents had a single sexual partner, whereas the rest (15.5%) had multiple sexual partners in their lifetime. Only twenty-one (7.4%) of women had history of drinking alcohol and chewing chat each. About, 26(9.2%) of respondents had history of sexual transmitted infection while only 1.4% of respondents had history of smoking cigarettes. Regarding HIV/AIDS, majority 223 (78.8) of respondents had negative sero-status while 20 (7.1) of them reported as positive sero-status (Table 2).

**Table 2:** Reproductive health and risk behavior related characteristics among eligible women attending maternal health services at Adare General Hospital, Hawassa city, Southern Ethiopia, 2022.

Variables	CCSS utilization		Total, n (%)
	Yes, n (%)	No, n (%)	
Parity			
≤ 1	8 (14.3)	44 (19.4)	52 (18.4)
2–4	43 (76.8)	133 (58.6)	176 (62.2)
≥ 5	5 (8.9)	50 (22.0)	55 (19.4)
Age at menarche (in years)			
≤ 12	9 (16.1)	41 (18.1)	50 (17.7)
> 12	47 (83.9)	186 (81.9)	233 (82.3)
Characteristics of menstruation			
Regular	36 (64.3)	177 (78.0)	213 (75.3)
Sometimes irregular	15 (26.8)	40 (17.6)	55 (19.4)
Always irregular	5 (8.9)	10 (4.4)	15 (5.3)
Modern contraception use			
Yes	45 (80.4)	148 (65.2)	193 (68.2)
No	11 (19.6)	79 (34.8)	90 (31.8)
Type of modern contraception used (n = 193)			
Oral contraceptive pills	19 (42.2)	30 (20.3)	49 (25.4)
Injectable	14 (31.1)	43 (29.1)	57 (29.5)
Implanon	7 (15.6)	56 (37.8)	63 (32.6)
IUCD	5 (11.1)	15 (10.1)	20 (10.4)
Jaddle	0 (0.0)	4 (2.7)	4 (2.1)
Age at first sexual debut (in years)			
< 18	33 (58.9%)	87 (38.3%)	120 (42.4%)
≥ 18	23 (41.1%)	140 (61.7%)	163 (57.6%)
Lifetime number of sexual partner			
Single	43 (76.8)	196 (86.3)	239 (84.5)
Multiple	13 (23.2)	31 (13.7)	44 (15.5)
History of drinking alcohol			
Yes	8 (14.3)	13 (5.7)	21 (7.4)
No	48 (85.7)	214 (94.3)	262 (92.6)
Have you ever chewing chat			
Yes	6 (10.7)	15 (6.6)	21 (7.4)
No	50 (89.3)	212 (93.4)	262 (92.6)
Have you ever smoked cigarette			
Yes	0 (0.0)	4 (1.8)	4 (1.4)
No	56 (100.0)	223 (98.2)	279 (98.6)
History of STI exposure			
Yes	5 (8.9)	21 (9.3)	26 (9.2)
No	51 (91.1)	206 (90.7)	257 (90.8)
Self-reported HIV status			
Positive	13 (23.2)	7 (3.1)	20 (7.1)
Negative	35 (62.5)	188 (82.8)	223 (78.8)
Unknown	8 (14.3)	32 (14.1)	40 (14.1)

### Knowledge and sources of information related characteristics of respondents

Among the total respondents, 225 (79.5%) of them heard about cervical cancer. The study sought to find the preferences for sources of information about cervical cancer from the respondents who indicated to have heard about it. Accordingly, the results show many of the respondents heard about cervical cancer form health personnel at 110 (38.9%) followed by Television/Radio at 82 (29.0%). About, 119 (42.0%) of women had known someone with CC and nearly, quarter (25.4%) of women had discussion about CC with healthcare providers.

**Figure 1:**
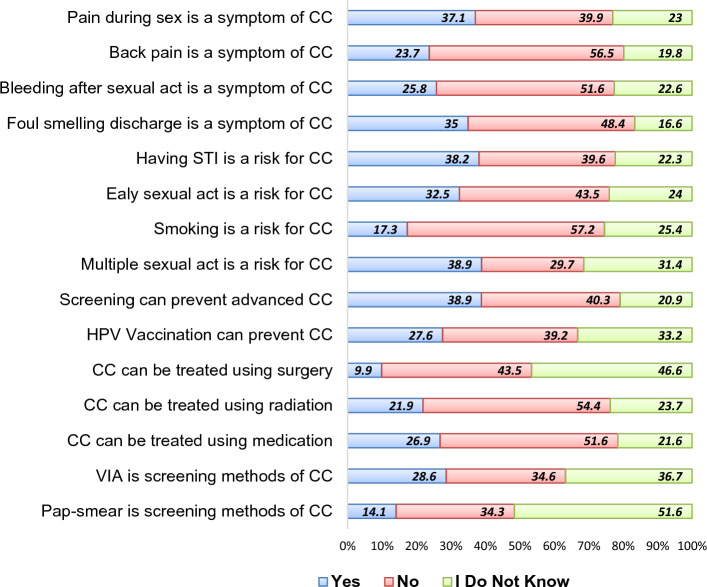
Main presenting symptoms, risk factors, prevention measures and treatment options of cervical cancer identified by women at Adare General Hospital, Hawassa city, Southern Ethiopia, 2022.

Respondents’ scores were combined, and the mean score was calculated in order to classify their comprehensive knowledge of cervical cancer. Accordingly, 120 (42.4%) of women had good comprehensive knowledge about CC (Table 3). Pain during sex is mentioned as symptoms of CC by 37.1% of women while multiple sexual partners (38.9%) was the most common risk factors of CC mentioned by women. Furthermore, medication and VIA was reported by 26.9% and 28.6% of women as means of treatments of CC and screening methods of CC, respectively (Fig. 1).

**Table 3:** Knowledge and sources of information related characteristics among eligible women attending maternal health services at Adare General Hospital, Hawassa city, Southern Ethiopia, 2022.

Variables	CCSS utilization		Total, n (%)
	Yes, n (%)	No, n (%)	
Ever heard of cervical cancer			
Yes	44 (78.6)	181 (79.9)	225 (79.5)
No	12 (21.4)	46 (20.3)	58 (20.3)
Source of information*			
Health professional	22 (39.3)	88 (38.8)	110 (38.9)
TV/radio	27 (48.2)	55 (24.2)	82 (29.0)
Friends	5 (8.9)	25 (11.0)	30 (10.6)
Family/relatives	6 (10.7)	16 (7.0)	22 (7.8)
Others	3 (5.4)	4 (1.8)	7 (2.5)
Know someone with cervical cancer			
Yes	40 (71.4)	79 (34.8)	119 (42.0)
No	16 (28.6)	148 (65.2)	164 (58.0)
Discussion about CC with healthcare provider			
Yes	26 (46.4)	46 (20.3)	72 (25.4)
No	30 (53.6)	181 (79.7)	211 (74.6)
Comprehensive knowledge			
Good	43 (76.8)	77 (33.9)	120 (42.4)
Poor	13 (23.2)	150 (66.1)	163 (57.6)

NB: * multiple responses were considered; thus, the sum might be more than respondents who indicated to have heard about cervical cancer.

### Magnitude of cervical cancer screening service utilization

Among eligible women interviewed, only 19.8% (95% CI 15.5%, 24.0%) of them had undertaken screening for cervical cancer at least once within the past five years. Out of screened, 37.5%, 17.9% and 12.5% were screened because of health care worker, husband/relatives and friends’ initiation respectively. However, 32.0% of respondents are self-initiated to do screening. Among respondents who undertake screening services, 36(64.3%) of them were screened before 3 years while 50(89.3%) of them were screened once in their life. About 52 (92.9%) of screened respondents were willing to undertake cervical cancer screening services again (Table 4).

**Table 4:** Magnitude of cervical cancer screening service utilization among eligible women attending maternal health services at Adare General Hospital, Hawassa city, Southern Ethiopia, 2022.

Variable	Frequency	Percentage (%)
Screened for cervical cancer at least once within the last 5 years		
Yes	56	19.8
No	227	80.2
Is there person who initiates you for cervical cancer screening		
Yes	38	67.9
No	18	32.1
Who initiates you		
Self	18	32.0
Health worker	21	37.5
Husband/relatives	10	17.9
Friends	7	12.5
Frequency of screening		
Once	50	89.3
More than once	6	10.7
Time since last screening		
Within 3 years	20	35.7
Before 3 years	36	64.3
CCS again		
Yes	52	92.9
No	4	7.1

### Reason for not getting screened for cervical cancer

**Figure 2:**
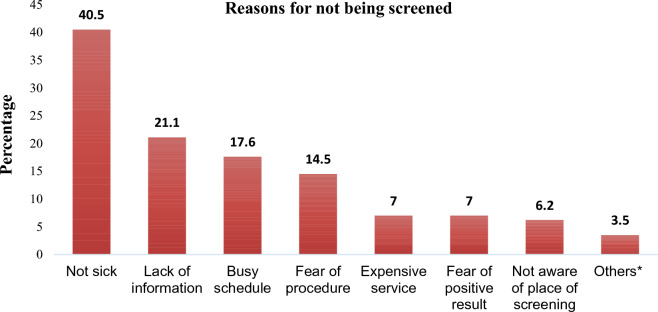
Reasons for not being screened mentioned by women attending maternal health service at Adare General Hospital, Hawassa city,  Southern Ethiopia, 2022.

Being healthy, lack of information, fear of procedure and fear of positive result were cited as the major reason for not being screened for cervical cancer in 40.5, 21.1%, 14.5% and 7.0% respectively by the respondents (Fig. 2).

### Factors associated with cervical cancer screening utilization

Using logistic regression analysis, the influences of several independent variables were examined on the utilization of CC screening. In the first step, bivariable logistic regression analysis was done to select candidate variable for multivariable logistic regression. Accordingly, residence, number of sexual partners, modern contraception use, comprehensive knowledge, knowing someone with cervical cancer and having discussion about CC with healthcare providers were significantly associated with utilization of CC screening service at *p* value of less than 0.25. Then, after controlling for the effect of other confounding variables; place of residence, modern contraception use, discursion about CC with healthcare provider and comprehensive knowledge about CC were remained significant factors of cervical cancer screening service utilization.

Women living in rural area were 63% less likely to be screened for CC than women living in urban area (adjusted OR = 0.37; 95% CI 0.14–0.96). Likewise, women who used modern contraception was 2.5 times more likely to had CC screening service utilization compared with their counterpart (adjusted OR = 2.49; 95% CI 1.04–5.96). Furthermore, having discussion about CC with healthcare provider increase the odds of screening by 2.3 times more likely as compared to women who had no discussion about CC with healthcare provider (adjusted OR = 2.34; 95% CI 1.08–5.07). Finally, women who had poor comprehensive knowledge about CC was 75% less likely to utilize CC screening service as compared to women who hand good comprehensive knowledge about CC (adjusted OR = 0.25; 95% CI 0.10–0.62) (Table 5).

**Table 5:** Bi-variable and multivariable logistic regression of factors associated with CC screening service utilization among eligible women attending maternal health services at Adare General Hospital, Hawassa city, Southern Ethiopia, 2022.

Variables	CCSS Utilization		COR (95% CI)	AOR (95% CI)
	Yes	No		
Age of respondent (in years)				
30–34	28	86	1	1
35–39	22	94	0.72 (0.38, 1.35)	1.01 (0.44, 2.33)
40–49	6	47	0.39 (0.15, 1.02)*	0.65 (0.19, 2.27)
Educational status				
No formal education	15	102	0.39 (0.19, 0.80)*	1.02 (0.40, 2.57)
Primary	11	49	0.60 (0.27, 1.34)*	2.60 (0.89, 7.61)
Secondary	7	15	1.24 (0.45, 3.42)	2.02 (0.60, 6.83)
College and above	23	61	1	1
Place of residence				
Urban	49	130	1	1
Rural	7	97	0.19 (0.08, 0.44)*	0.37 (0.14, 0.96)**
Number of sexual partners				
1	43	196	1	1
≥ 1	13	31	1.91 (0.92, 3.95)*	1.67 (0.67, 4.15)
Modern contraception use				
Yes	45	159	2.18 (1.07, 4.46)*	2.49 (1.04, 5.96)**
No	11	68	1	1
Know someone with cervical cancer				
Yes	40 (71.4)	79 (34.8)	4.68 (2.47, 8.89)	1.99 (0.79, 5.03)
No	16 (28.6)	148 (65.2)	1	1
Discussion about CC with healthcare provider				
Yes	26	46	3.41 (1.84, 6.32)	2.34 (1.08, 5.07)**
No	30	181	1	1
Comprehensive knowledge about cervical cancer				
Good	43	77	1	1
Poor	13	150	0.16 (0.08, 0.31)*	0.25 (0.10, 0.62)**

Hosmer–Lemeshow goodness-of-ft = 0.069.

NB. 1: reference, *Remained significant at *P* value < 0.25, **Remained significant at *P* value < 0.05.

## Discussion

The aim of the study was to assess the magnitude and associated factors of cervical cancer screening service utilization among women aged 30 to 49 who were receiving maternal health services at Adare General Hospital in southern Ethiopia. In this regard, the findings of this study showed about 56 (19.8%) of women were received cervical cancer screening at least once within the last five years. This magnitude was in line with reports of studies conducted in Nigeria 15.6%^20^, Rwanda 23%^21^, Kenya 17.5%^22^, and different parts of Ethiopia; 19.5% in Bahirdar^23^, 21.2% in North Shoa^24^, 22.9% in Wolyita zone^14^. However, the magnitude of this study is higher than studies conducted in India; 11.62%^25^, Pakistan; 5.9%^26^, Burkina Faso; 11.7%^27^, Uganda; 4.8%^28^. It is also higher compared with previous findings reported in different parts of Ethiopia; 3% in Finote Selam town^29^, (12.2% in St. Paul’s Teaching and Referral Hospital^30^, 8.7% in Ambo town^31^, 5.9% in Arba Minch Town^32^. The possible explanation for this inconsistency might be variations in the measurement of the outcome variable, socio-demographic characteristics, study period, types of tests utilized, study setting and cultural background and taboos regarding reproductive issues. For example, study participants in India reported “due to custom and cultural boundaries we are not allowed to go outside alone without any male members of the family”.

In contrast, the magnitude found in this study was lower than those reported in China; 47%^33^, Korea; 46%^34^, Thailand; 64.9%^35^, South Africa; 41.4%^36^, Kenya; 46%^37^, and Mettu Karl referral hospital; 29.9%^38^.

This discrepancy might be due to the difference types of study participant involved, health-seeking behavior of the study participants, and level of attention given to cancer screening programmes present in the country. For example, a study participants of a study conducted in China^33^ were women with HIV. As a result, CC screening service utilization might be increased. This explanation was supported by a study done in North Shoa^24^, which reported that having positive HIV status increases CC screening utilization by more than sixteen times compared with having negative HIV status. Furthermore, the above-mentioned countries as well as facilities might have good community awareness and healthcare provider capacity building programmes compared with the current study setting.

Other aim of this study was establishing association between independent variables with the dependent variables. As a result, place of residence, modern contraception use, discursion about CC with healthcare provider and level of comprehensive knowledge about CC were significantly associated with CC screening service utilization.

This study found that place of residence was one of significantly associated factors with utilization of CC screening. Being dweller of rural residence were 63% less likely to be screened for CC compared with dweller of urban residence. The finding matches with previous studies conducted in Nepal^39^, Kenya^40^, Amhara region^41^, St. Paul’s Teaching and Referral Hospital^30^ and southern Ethiopia^42^. The possible justification might be due to limited awareness and lower educational status of the rural women. Furthermore, the reason for this might be women who lives in rural area may not have better access to the service as well as information about cervical cancer and its screening. This suggests the need to focus on strengthening of reproductive health programs particularly cervical cancer prevention by integrating with health extension programme in rural area is mandatory.

In the final logistic regression model, modern contraception use was found to be a significant factor of CC screening utilization. Women who were used modern contraception were more than two times more likely to be screened for cervical cancer as compared to their counterparts. This evidence is in line with previous study done in in Ouagadougou, Burkina Faso^27^, Mettu Karl referral hospital, south west Ethiopia^38^, and Ambo town, central Ethiopia^43^. The possible explanation for this association might be women who used modern contraceptive may get additional information about cervical cancer and its screening at the time of taking family planning services. Making frequent contact with healthcare providers than non-user of modern contraceptive might be other possible reason.

This tells us the necessity of integrating CC screening service with different maternal health services as it increases service utilization.

Discussion about cervical cancer with healthcare provider was also found to be associated factors of CC screening utilization in this study. Having discussions about cervical cancer with a healthcare provider increases the odds of CC screening utilization by more than two times compared with women without discussions about cervical cancer with a healthcare provider. This association was consistent with former studies done in China^33^, Thailand^35^, and different parts of Ethiopia; Dire Dawa^19^, Ambo town^31^, Jimma town^16^, which showed that making discussion about CC with healthcare provider increases utilization of CC screening. This evidence also supported by a study conducted in USA^44^, Uganda^28^, and Kenya^37^, which revealed that having got recommendation by healthcare providers were more likely to increase CC screening utilization compared with women who had no recommendation by healthcare providers. The possible reason for this finding might be due to women who have made discussion with health care providers may get adequate information about the disease, benefit of screening, methods of screening and place of screening. Additionally, the information delivered by healthcare provider might increase awareness about CC and trusted. This highlights the important role that healthcare provider plays in the acceptance of service utilization. Therefore, should be undertaken for healthcare providers to increase adequate information given to the customers, which increases CC screening service utilization.

Moreover, in this study, comprehensive knowledge about CC was significantly associated with CC screening utilization. Women who had poor comprehensive knowledge about CC were 75% less likely to report utilization of CC screening service than women who had good comprehensive knowledge about CC. This result is in agreement with other studies conducted in Japan^45^, Northern district of Yangon, Myanmar^46^, Rwanda^21^, Kenya^22^, and several parts of Ethiopia; Tigray region^47^, Amhara region^41^, Dire Dawa^19^, Ambo town^31^, and Jimma town^16^. This might be due to having good comprehensive knowledge about cervical cancer may increase awareness and the interest of women to take part in CC screening service and hence, increases service utilization.

Despite the study includes at risk population as study participants, it is not without weakness. Thus, it could be necessary to comprehend the following constraints when evaluating results. First, there is no system in place to prevent recall bias from influencing participants' responses. Second, because the study is cross-sectional, it is challenging to show a causal relationship between the independent and outcome variables. Thirdly, it may not be suitable to represent the entire population accurately in this facility-based study. Lastly, statistical power and generalization may be compromised due to small sample size utilized in this study.

## Conclusions

The study found nearly one out of five of women had utilized CC screening service at least once within 5 years. In this study, place of residence, modern contraception use, discursion about CC with healthcare provider and level of comprehensive knowledge about CC were determining factors to utilize CC screening service. Thus, to increase the utilization of CC screening service among eligible women regularly, it is better to create awareness programs for early detection by improving educational interventions that teach subject matter of cervical cancer to increase women’s attitude towards screening. Additionally, discussion and improving the confidence of women by health care providers to undergo screening is suggested. Furthermore, strengthening service linkage among departments and giving short and long training for healthcare provider to increase delivery of information for the vulnerable group is also recommended.

## Data Availability

All relevant materials and data supporting the findings of this study are not available for online access, however readers who wish to gain access to the data can write to the corresponding author Abiyu Ayalew Assefa at abiyman143@gmail.com.

## Abbreviations

AOR: Adjusted odd ratio
CI: Confidence interval
OR: Odds ratio
COR: Crude odd ratio
SPSS: Statistical package for the social sciences
VIA: Visual inspection of acetic acid
EFY: Ethiopian fiscal year
CC: Cervical cancer
